# Female Genital Schistosomiasis Lesion Resolution Post-Treatment with Praziquantel in Zambian Adults

**DOI:** 10.4269/ajtmh.23-0552

**Published:** 2024-01-09

**Authors:** Chishiba Kabengele, Sepo Mwangelwa, William Kilembe, Bellington Vwalika, Mubiana Inambao, Vernon Moonga, Constance Himukumbwa, W. Evan Secor, Rachel Parker, Amanda Tichacek, Amaya L. Bustinduy, Susan Allen, Kristin M. Wall

**Affiliations:** ^1^ Center for Family Health Research Zambia, Lusaka, Zambia;; ^2^ Center for Family Health Research Zambia, Ndola, Zambia;; ^3^ Centers for Disease Control and Prevention, Atlanta, Georgia;; ^4^ Rwanda Zambia HIV Research Group, Department of Pathology & Laboratory Medicine, School of Medicine and Hubert Department of Global Health, Rollins School of Public Health, Laney Graduate School, Emory University, Atlanta, Georgia;; ^5^ Department of Clinical Research, London School of Hygiene & Tropical Medicine, London, United Kingdom

## Abstract

We evaluated changes in female genital schistosomiasis (FGS) 6 to 12 months after praziquantel treatment among 43 adult Zambian women. Most women (60%) experienced decreased FGS severity and 23% experienced complete lesion resolution. This is the first study to demonstrate a meaningful effect of praziquantel treatment of FGS in adult women.

## INTRODUCTION

Infection with *Schistosoma haematobium* causes urogenital schistosomiasis, which can cause female genital schistosomiasis (FGS). Female genital schistosomiasis affects 56 million women and girls in Africa alone, and is one of the most neglected tropic diseases worldwide.^1^ Female genital schistosomiasis is associated with infertility, pregnancy complications, lost productivity, stigma,^2^ and HIV risk.^3^^,^^4^

There is uncertainty about whether praziquantel treatment of adult women with FGS helps to resolve FGS lesions.^2^^,^^5^^–^^9^ Few prior studies have explored this research question. From 2020 to 2021, the Center for Family Health Research Zambia (CFHRZ) evaluated the frequency and factors associated with post-treatment FGS lesion resolution among adult women.

## MATERIALS AND METHODS

### Baseline participant selection.

We recruited 497 participants from a prospective cohort of HIV-uninfected women at high risk for HIV/sexually transmitted infection (STI) in Lusaka and Ndola^10^ (two large, urban areas in Zambia with low schistosomiasis prevalence^11^) between March 2020 and December 2021. Women were female sex workers or single mothers ≥ 18 years old referred to CFHRZ research sites after community outreach at sex worker hotspots or postnatal clinics, respectively.

### Follow-up participant selection.

Women from the baseline cohort were invited to return to the research sites to evaluate FGS lesion resolution. Inclusion criteria were women diagnosed with FGS at baseline who received directly observed praziquantel treatment (40 mg/kg) 6 to 12 months earlier and who were not menstruating. Participants were enrolled until a target (based on available resources) of 40 to 45 women was reached.

### Survey procedures.

At CFHRZ research sites in Lusaka and Ndola, participants completed baseline surveys in the local language (Nyanja in Lusaka and Bemba in Ndola) to assess demographics; reproductive, gynecological, and urinary history and symptoms; and environmental exposures. Follow-up surveys ascertained symptoms and reinfection risk since treatment.

### Clinical procedures at baseline and follow-up.

During colposcopy (Bovie Colpo-Master™ CS-105LEDI Swing Arm Colposcope, Bovie, NY; continuous zoom ratio, 1:6.7 [0.67×–4.5×]; 3.9–27× magnification), endocervical and vaginal swabs were collected. Genital examination assessed inflammation, contact bleeding, discharge, ulceration, and adenopathy. The cervix was then cleared of any discharge, and photographs were taken prior to and after visual inspection with acetic acid (VIA). Four CFHRZ research doctors and nurses were trained by B. V. (an FGS co-author of *Female Genital Schistosomiasis: A Pocket Atlas for Clinical Health-Care Professionals*^12^) and M. I. to perform genital examinations, colposcopies, and VIAs, and to take photographs of the cervix.

Women provided urine samples for hematuria testing and urine filtration for detection of *S. haematobium* eggs by trained laboratory technicians. Participants were tested for gonorrhea, *Chlamydia*, high-risk human papilloma virus (hrHPV), trichomoniasis, *Candida*, bacterial vaginosis, syphilis, and HIV by trained laboratory technicians, as described elsewhere.^4^^,^^10^

### Female genital schistosomiasis diagnosis.

Colposcopy images were downloaded onto a computer for storage. All images were reviewed independently by B. V. and M. I. The standard FGS case definition (i.e., presence of any indicator: grainy sandy patches, homogenous yellow sandy patches, abnormal blood vessels, rubbery papules) was used.^12^ A score of 0 to 8 points was assigned to each participant based on the total number of FGS indicators observed and the number of cervical quadrants involved. In the event of disagreement, a third independent reviewer served as a tiebreaker. To reduce bias, gynecological examination, laboratory, and survey findings were unknown to the reviewers.

### Treatments and referrals.

Women with any FGS indicator, egg excretion, or hematuria were treated for free at our research site with praziquantel (40 mg/kg). Women diagnosed with STIs or vaginal dysbiosis were treated for free at our research facility per Zambian national guidelines. Women who were HIV, VIA, or hrHPV positive were referred for care per Zambian national guidelines.

### Data collection and management.

Survey data were collected on tablets using SurveyCTO (Dobility, Inc., Cambridge, MA). Clinical and laboratory data were collected on paper forms that were later entered into SurveyCTO. Data were imported weekly from SurveyCTO into MS Access (Microsoft Corporation, Redmund, WA) for long-term storage, quality control, and cleaning.

### Data analysis.

Analyses were conducted with SAS version 9.4 (SAS Institute, Cary, NC). *P* values are two tailed. Percent agreement and Cohen’s κ were calculated to assess interrater agreement of FGS diagnosis post-treatment.

In this study, we defined FGS lesion severity as the sum of the number of FGS indicators and cervical lesion locations. Variables hypothesized to be associated with FGS lesion resolution post-treatment included age, months since treatment, FGS lesion type, number of FGS indicators, number of cervical lesion locations, FGS severity, VIA results, contact bleeding, eggs in urine, STIs, vaginal dysbiosis, and environmental exposures since treatment. Variables were described using counts and percentages for categorical variables, and medians and interquartile ranges (IQRs) for continuous variables, overall and stratified by lesion resolution, with statistical differences quantified using χ^2^ or Fisher’s exact tests or Wilcoxon two-sample tests, as appropriate.

Last, we explored whether symptoms and gynecological examination findings changed pre- and post-treatment using descriptive statistics, with statistical differences quantified using McNemar’s test for paired data.

## RESULTS

We enrolled 43 women in follow-up (*n =* 18 from Lusaka, *n =* 25 from Ndola). Median participant age was 29 years (IQR = 6 years) with a median of 9 months (IQR = 5 months) between treatment and follow-up (Table 1).

**Table 1:** Baseline descriptive statistics overall and by FGS lesion resolution post-treatment

Variable	Total (*N* = 43)	FGS lesions resolved post-treatment (*n* = 10)	FGS lesions unresolved post-treatment (*n* = 33)*	*P* value†
Age, years; median (IQR)	29.0 (6.0)	29.5 (8)	29.0 (5.0)	0.954
Months since treatment, median (IQR)	9.2 (4.7)	8.1 (4.8)	9.2 (4.4)	0.476
FGS lesion type versus other type				
Abnormal blood vessels, *n* (%)	33 (77)	5 (50)	28 (85)	0.036
Homogenous yellow sandy patches, *n* (%)	26 (60)	6 (60)	20 (61)	1.000
Rubbery papules, *n* (%)	2 (5)	0 (0)	2 (6)	1.000
Grainy sandy patches, *n* (%)	9 (21)	2 (20)	7 (21)	1.000
No. of FGS indicators, median (IQR)‡	2.0 (1.0)	1.0 (1.0)	2.0 (1.0)	0.062
No. of cervical quadrants with FGS involvement, median (IQR)§	3.0 (2.0)	2.0 (1.0)	3.0 (2.0)	0.070
Severity score, points; median (IQR)‖	5.0 (3.0)	3.0 (1.0)	5.0 (3.0)	0.041
VIA status, *n* (%)				
Positive	20 (47)	7 (70)	13 (39)	0.148
Negative	23 (53)	3 (30)	20 (61)	
Contact bleeding, *n* (%)				
Yes	11 (26)	3 (30)	8 (24)	0.698
No	32 (74)	7 (70)	25 (76)	
Eggs in urine, *n* (%)				
Yes	1 (2)	0 (0)	1 (3)	1.000
No	42 (98)	10 (100)	32 (97)	
Baseline STI, *n* (%)¶				
Positive	18 (42)	5 (50)	13 (39)	0.717
Negative	25 (58)	5 (50)	20 (61)	

FGS = female genital schistosomiasis; IQR = interquartile range; STI = sexually transmitted infection; VIA = visual inspection with acetic acid.

* The 33 women who did not experience complete FGS lesion resolution included 16 who showed a decrease in lesion severity and 17 who did not.

† *P* values from χ^2^ or Fisher’s exact test for categorical variables and the Wilcoxon two-sample test for continuous variables.

‡ Grainy sandy patches, homogenous yellow sandy patches, abnormal blood vessels, and rubbery papules.

§ Cervical quadrants 1 through 4.

‖ Severity score = Number of FGS indicators + Number of lesion locations.

¶ *Chlamydia*, gonorrhea, trichomoniasis, and high-risk human papilloma virus.

**Figure 1.**
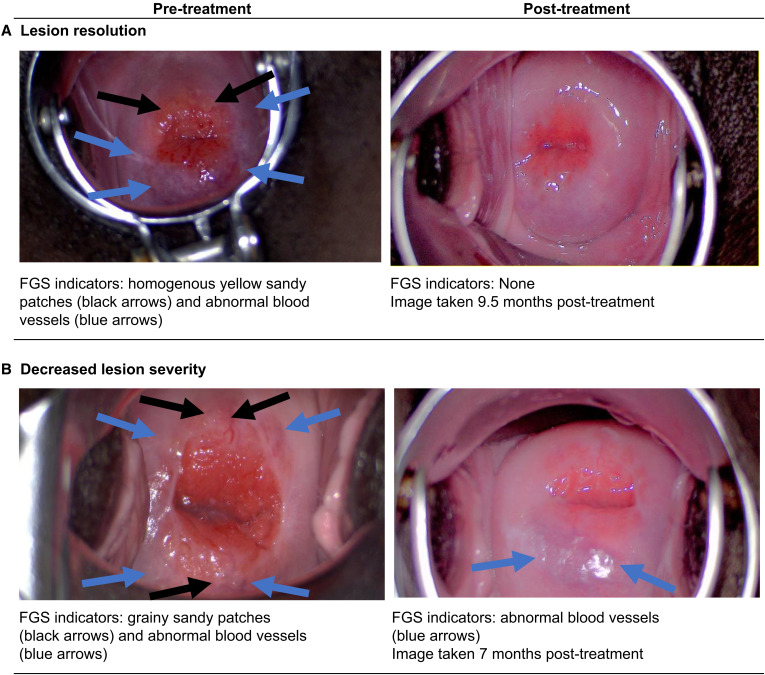
Representative images taken during colposcopy before and after praziquantel treatment of women experiencing (**A**) female genital schistosomiasis lesion resolution or (**B**) decreased lesion severity.

Primary reviewer agreement was 81% (Cohen’s κ = 0.6, indicating substantial agreement). Eight cases of disagreement were scored FGS positive by the third reviewer. After this adjudication, 60% of women (*n =* 26) showed a decrease in lesion severity or complete resolution, and 23% (*n =* 10 of the 26) demonstrated complete resolution post-treatment compared with their pretreatment evaluations. The remaining 17 women did not show a decrease in lesion severity or complete resolution. Representative pre- and post-treatment images showing decreased lesion severity and FGS lesion resolution are shown in Figure 1.

At baseline, 19 women had only 1 indicator (10 had abnormal blood vessels, 3 had grainy sandy patches, and 6 had homogenous yellow sandy patches); 13 had homogenous yellow sandy patches and abnormal blood vessels; 7 of 43 had grainy sandy patches and abnormal blood vessels; 1 had homogenous yellow sandy patches, abnormal blood vessels, and rubbery papules; 1 had grainy sandy patches, abnormal blood vessels, and rubbery papules; 1 had grainy sandy patches and abnormal blood vessels; and 1 had grainy sandy patches, homogenous yellow sandy patches, and abnormal blood vessels (Table 1).

Comparison groups in Table 1 are comprised of the 10 women who showed complete FGS lesion resolution versus the 33 whose lesions did not resolve completely (including 16 who showed a decrease in lesion severity and 17 who did not show a decrease in lesion severity). Less severe FGS at baseline was associated with lesion resolution (median baseline severity scores of 3 points [IQR = 1 point] versus 5 points [IQR = 3 points] for those whose lesions did and did not resolve, respectively; *P* = 0.041). Baseline STI status was not associated with changes in FGS lesions (Table 1). Self-reported symptoms and gynecological examination findings were rare and not statistically significantly different pre- and post-treatment; however, decreases in self-reported incontinence (21% versus 12%) and inflammation of the cervix on gynecological examination (17% versus 8%) were noted (data not shown).

## DISCUSSION

After praziquantel treatment, 26 of 43 (60%) women who had FGS at baseline experienced decreases in lesion severity, and 10 of 43 (23%) experienced complete FGS resolution. Having less severe baseline disease was associated with FGS lesion resolution post-treatment. Few studies have explored this research issue, and no prior studies have shown high frequencies of FGS lesion resolution by colposcopy in adult women post-treatment.^2^

Richter et al.^9^ showed that among 21 women with FGS in southern Malawi, symptoms such as macrohematuria, dysuria, backache, and lower abdominal pain subsided in most patients 2 to 9 weeks after treatment with praziquantel. Among the nine women with FGS who were reexamined, lower reproductive tract abnormalities were at least partially reversible.

By contrast, Kjetland et al.^6^ evaluated FGS lesion resolution 3 and 12 months after initial treatment with praziquantel in women age 20 to 49 years in rural Zimbabwe. Although urinary ova excretion decreased, neither genital lesions nor contact bleeding decreased significantly after treatment. However, a subsequent study^7^ in this same cohort found that a history of praziquantel treatment before age 20 was associated significantly with the absence of sandy patches and contact bleeding.

A possible explanation for the discrepancy between our findings and the Zimbabwean studies is our urban setting, where exposure to reinfection is limited. Participants’ exposure history indicated that many were likely exposed during travel to rural areas or when living in rural areas as children, and only one participant reported a potential new exposure after treatment. By contrast, the Zimbabwean women lived in *S. haematobium*–endemic rural areas where the likelihood of repeat exposure is high. It is possible that FGS lesions are more severe with repeated exposures, reducing the effectiveness of treatment. Thus, not only should women receive treatment to reduce the severity of FGS, but optimizing the dosing frequency should be explored. In addition, our study participants were receiving regular screening and treatment of STIs, bacterial vaginosis, and *Candida*, and therefore may respond to FGS treatment differently than women with untreated gynecological disturbances.

Our data indicate that treatment of adult women provides measurable benefits to reduce or resolve FGS. However, most FGS programs are focused on school-age children, leaving older girls and women at risk. To reach women no longer in school, the impact of integrating FGS screening and treatment programs into existing STI or family planning services could be explored.

Some limitations warrant consideration. Error in FGS diagnosis is possible. However, reviewers agreed on 81% of reviews, indicating a greater level of agreement than observed in previous studies. For example, a different study in Zambia^13^ found a Cohen’s κ of 0.16, indicating slight agreement. In addition, our study population was not selected randomly. However, bias is unlikely because selection was not associated with both baseline lesion severity and the outcome of resolution. Although we cannot rule out that some lesions were new and caused by reinfection, only one participant reported a possible exposure since treatment, and no women had eggs in their urine at follow-up, suggesting a limited role for reinfection in this urban population. Last, our sample size was limited. A robust study of praziquantel effectiveness to treat FGS is needed urgently.

## CONCLUSION

This is the first study to demonstrate a sizable effect of praziquantel to treat adult women for FGS. These data advocate for treatment programs for this population.
